# Noise correlation in PET, CT, SPECT and PET/CT data evaluated using autocorrelation function: a phantom study on data, reconstructed using FBP and OSEM

**DOI:** 10.1186/1471-2342-5-5

**Published:** 2005-08-25

**Authors:** Pasha Razifar, Mattias Sandström, Harald Schnieder, Bengt Långström, Enn Maripuu, Ewert Bengtsson, Mats Bergström

**Affiliations:** 1 Uppsala University, Centre for Image Analysis, Lägerhyddsv. 3, SE-752 37 Uppsala, Sweden; 2 Uppsala Imanet AB, Box 967, SE-751 09 Uppsala, Sweden; 3 Uppsala University Hospital, Department of Hospital Physics, SE-751 85 Uppsala, Sweden; 4 Department of Pharmaceutical Biosciences, Uppsala University, Uppsala, Sweden

## Abstract

**Background:**

Positron Emission Tomography (PET), Computed Tomography (CT), PET/CT and Single Photon Emission Tomography (SPECT) are non-invasive imaging tools used for creating two dimensional (2D) cross section images of three dimensional (3D) objects. PET and SPECT have the potential of providing functional or biochemical information by measuring distribution and kinetics of radiolabelled molecules, whereas CT visualizes X-ray density in tissues in the body. PET/CT provides fused images representing both functional and anatomical information with better precision in localization than PET alone.

Images generated by these types of techniques are generally noisy, thereby impairing the imaging potential and affecting the precision in quantitative values derived from the images. It is crucial to explore and understand the properties of noise in these imaging techniques. Here we used autocorrelation function (ACF) specifically to describe noise correlation and its non-isotropic behaviour in experimentally generated images of PET, CT, PET/CT and SPECT.

**Methods:**

Experiments were performed using phantoms with different shapes. In PET and PET/CT studies, data were acquired in 2D acquisition mode and reconstructed by both analytical filter back projection (FBP) and iterative, ordered subsets expectation maximisation (OSEM) methods. In the PET/CT studies, different magnitudes of X-ray dose in the transmission were employed by using different mA settings for the X-ray tube. In the CT studies, data were acquired using different slice thickness with and without applied dose reduction function and the images were reconstructed by FBP. SPECT studies were performed in 2D, reconstructed using FBP and OSEM, using post 3D filtering. ACF images were generated from the primary images, and profiles across the ACF images were used to describe the noise correlation in different directions. The variance of noise across the images was visualised as images and with profiles across these images.

**Results:**

The most important finding was that the pattern of noise correlation is rotation symmetric or isotropic, independent of object shape in PET and PET/CT images reconstructed using the iterative method. This is, however, not the case in FBP images when the shape of phantom is not circular. Also CT images reconstructed using FBP show the same non-isotropic pattern independent of slice thickness and utilization of care dose function. SPECT images show an isotropic correlation of the noise independent of object shape or applied reconstruction algorithm. Noise in PET/CT images was identical independent of the applied X-ray dose in the transmission part (CT), indicating that the noise from transmission with the applied doses does not propagate into the PET images showing that the noise from the emission part is dominant. The results indicate that in human studies it is possible to utilize a low dose in transmission part while maintaining the noise behaviour and the quality of the images.

**Conclusion:**

The combined effect of noise correlation for asymmetric objects and a varying noise variance across the image field significantly complicates the interpretation of the images when statistical methods are used, such as with statistical estimates of precision in average values, use of statistical parametric mapping methods and principal component analysis. Hence it is recommended that iterative reconstruction methods are used for such applications. However, it is possible to calculate the noise analytically in images reconstructed by FBP, while it is not possible to do the same calculation in images reconstructed by iterative methods. Therefore for performing statistical methods of analysis which depend on knowing the noise, FBP would be preferred.

## Background

Computed tomography (CT) is a technique based on measurement of X-ray transmission through the object to provide visible thin slices through any section in a human; in other words, it is a technique for creating two dimensional (2D) cross section images of three dimensional (3D) objects [1]. Positron Emission Tomography (PET) and Single Photon Emission Tomography (SPECT) are built on the concept of CT, but use tracing of molecules labelled with positron and gamma ray emitting radionuclides, respectively, to illustrate metabolic and physiological activities in certain organs and tissues. PET/CT combines two state-of-the-art imaging modalities: PET and CT. PET provides high sensitivity functional information of lesions in the body while CT provides detailed information about the location, size, and shape of these lesions, but cannot differentiate pathological lesions from normal structures with the same sensitivity as PET [2-5]. The combined PET/CT scanner has proved to increase the diagnostic value compared to each modality used separately [6].

In PET/CT and SPECT/CT, the CT data are, except for imaging and anatomical delineation, used as transmission data for performing attenuation correction during the reconstruction. PET, SPECT and PET/CT data are usually reconstructed either analytically by filtered back projection (FBP) or iteratively by for instance, ordered subsets expectation maximisation (OSEM), in SPECT followed by 3D filtering. OSEM needs less computation time compared with earlier versions of iterative reconstructions such as maximum likelihood expectation maximisation (ML-EM) [7]. CT data are reconstructed using FBP with corrections for cone beam geometry and filtered using standard filters related to the imaging task.

In PET and PET/CT the convolution kernel applied on projections in an FBP algorithm is a combination of a ramp filter to cope with blurriness of the image after back projecting the projections [8,9] and a low-pass filter e.g. Hanning filter to damp the high frequency behaviour of a ramp filter. FBP is a relatively fast process but it has the drawback that the images are noisier and more sensitive to disturbing factors e.g. patient movements during the scan and in PET studies, between transmission and emission scans, leading sometimes to degradation of image quality.

In SPECT it has been shown that OSEM gives fewer artefacts compared to FBP [10,11]. It has also been shown that changing the detector head orbit from circular to elliptical may improve the isotropy of recovered resolution [12].

One limiting factor with respect to the visualisation of discrete changes, e.g. as induced in pathological lesions is noise in the images. This noise primarily comes from the inherent random variations in the counting of photons and is related to the number of photons detected and used for the generation of images. In CT a high flux of photons is used, giving rise to noise in the images which is about 0.1%, whereas PET and SPECT with fewer photons have noise levels more typically of about 10%.

The main sources of noise in PET images are in decreasing order of magnitude: emission, transmission and blank scans [13]. Detectors, electronics and recorder systems together may add to the noise [14,15]. The choice of reconstruction algorithm and type of convolution kernel used in the reconstruction algorithm significantly affect the magnitude and texture of noise. Other factors which contribute to noise features include mode of attenuation correction and other types of corrections e.g. for variation in detector uniformity, random coincidences, scattered radiation and compensation for radioactive decay as discussed by Alpert et al [16].

Wilson [17] has shown that the magnitude (variance) of noise spreads from regions with higher signal magnitude towards regions with lower signal in FBP reconstruction, but not in iterative reconstruction. Riddell et al [18] has shown that FBP provides a uniform and intensity independent noise distribution over the whole reconstructed image with very low variation within the image even when signal intensity varied significantly from one region to another. In contrast OSEM provides an intensity dependent noise within the image with the noise magnitude being higher in regions with higher intensity compared to the regions with lower intensity. OSEM provides better signal-to-noise ratios (SNR) in regions with high and low intensity compared to FBP yet more dramatic improvement in SNR in regions with low intensity.

Studies have shown that in images reconstructed using OSEM and a low number of iterations, the noise is correlated at shorter distances compared to images reconstructed by FBP. In another study has been shown that images reconstructed using FBP, correlation of each image element influences 1 or 2, pixels of the nearest neighbors in PET [19].

CT has an advantage compared to the two nuclear techniques in its low structural noise in the images. This capacity of low noise and efficient dose utilisation has enabled this technique to visualize low contrast objects [9]. Modern CT equipment has the ability to apply a care dose function, with an automated reduction of the dose for non-circular patient cross-sections based on a reduction of the intensity of X-ray tube current at the angular positions at which the patient diameter is smallest. This procedure is performed online with dose regulation during the scanning with preservation of the image quality and noise magnitude [20-23].

Although different aspects of noise have been covered extensively in the literature, we still feel that one aspect has not been adequately covered: the angular dependence of noise correlation in cases when the investigated object is asymmetrical. With asymmetric objects, the count rates will be different in the different acquisition angles and the relative magnitude of the noise will therefore be different. It is possible that this angular-dependent noise would in the image reconstruction propagate to the images and there generate a noise correlation which is non-isotropic.

In our previous study we explored the pattern of noise correlation in experimentally generated PET images acquired in 2D and 3D mode and reconstructed using FBP and OSEM, with emphasis on the angular dependence of correlation, and evaluated using the autocorrelation function (ACF) [24]. In our present work we extend these observations to other tomographic imaging modalities by applying autocorrelation function on PET, CT, PET/CT and SPECT images reconstructed using FBP and OSEM.

## Methods

The PET experiments were performed on an ECAT Exact HR+ PET camera (CTI/Siemens, Knoxville, Tennessee) [25]. This unit contains 32 detector rings separated by removable lead septa, and is capable of performing 2D and 3D data acquisition with an axial field of view (FOV) of 15.5 cm and spatial resolution of 5.1–5.4 mm. The system generates 63, 2D images with a matrix of 128 × 128 pixels. The CT experiments were performed on a Siemens Somatom Sensation 16 CT scanner (Siemens, Erlangen, Germany). This scanner acquires 16 slices/rotation with a rotation time of 0.5 s. Images were reconstructed into 512 × 512 matrices; maximum image FOV is 50 cm. The SPECT measurements were made with Millennium VG dual-headed gamma camera with 5/8 "NaI (TI) detectors and a Hawkeye X-ray tomography for attenuation correction (AC) and anatomical information (General Electric Medical Systems, Haifa, Israel). The image matrix was 128 × 128 pixels.

PET/CT experiments were performed employing Discovery ST (D-ST) (GE medical Systems). The scanner combines a helical multi-slice High Speed Ultra 16 slice, CT scanner and a multi-ring BGO block detector PET tomography, which are arranged in 24 rings, spaced by 3.27 mm covering an axial FOV of 157 mm with different spatial resolution depending on the distance from the centre of FOV. The scanner creates 47 128 × 128 fused images [26].

In the PET study, the radionuclides ^11^C and ^68^Ga with 20.3 min and 67.6 min half-lives, respectively, were used. ^11^C was produced using a Scanditronix MC-17 cyclotron (Scanditronix AB, Uppsala, Sweden). ^68^Ga was obtained from a ^68^Ge generator [27] In the SPECT studies the radionuclide ^99 m^TC with 6.02 h was used. ^99 m^Tc was eluted from a ^99^Mo/^99 m^Tc generator from Mallinckrodt.

All experiments with each one of the modalities were performed on either a 20-cm-long elliptical torso phantom with 30-cm-diameter long axis and 20-cm short axis or a cylindrical water-filled Nema phantom with 20-cm diameter and 20-cm length [28].

Prior to each PET experiment, a 60-min blank scan was performed with rotating ^68^Ge/^68^Ga rod sources without any phantom in the gantry. Then, a 10-min transmission scan was performed where the respective phantom without radioactivity was placed at the centre of the FOV of the scanner. The phantoms were filled with 80 MBq of either ^11^C or ^68^Ga and 30-min emission scans in 2D mode were made. Finally, to avoid artefacts in the images caused by movement of the object between transmission and emission scan, a 10-min post-injection ('hot') transmission scan was performed. The SPECT emission acquisitions were made with 60 × 50-s views on phantoms that were filled with 50 MBq of ^99 m^Tc. The used energy window was 140 keV ± 10%. The transmission used for attenuation correction was made with a Hawkeye CT put on half rotation, 140 kVp and 3.0 mA.

In the CT studies the phantoms were filled with water and the tomography was set to create both two- and three-mm-thick slices. Different numbers of slices were obtained in each scan depending on the desired thickness of slices. Each experiment was additionally performed applying the care dose function. In PET/CT, the study was performed on phantoms filled with 60 MBq ^68^Ga in the Nema and 80 MBq ^68^Ga in the Torso phantom. The scans were started by transmission (CT) scans using 140 kVp, 10 mA, 30 mA and 100 mA setting of the X-ray tube followed by 12 × 30-min emissions.

The PET images reconstructed using the initial transmission scan for attenuation correction were not used because of slight observed positioning errors when replacing the phantom after filling it with radioactivity. Instead a weighted segmentation technique described elsewhere [29], as included in the ECAT 7.2 software (CTI, Knoxville, Tennesse), was applied to the hot transmission data prior to its use for attenuation correction.

In PET and PET/CT, both FBP and attenuation-weighted OSEM as included in the scanner software were used for reconstructing the images. Different types of filters can be used with each of the reconstruction methods. A Gausssian filter with size of 6 mm (FWHM) was used in this study for reconstruction of images using FBP and OSEM. Also same number of subsets and iterations in OSEM was used for reconstruction of data in PET and PET/CT studies. PET/CT images were reconstructed with three different combinations of CT transmission data (10, 30 and 100 mA) and emission data. All CT images were reconstructed using FBP with corrections for cone beam geometry and filtered using standard abdomen filter, as included in the scanner software. All SPECT images were reconstructed using both FBP and OSEM algorithms, as included in the Entegra workstation, using the default settings for filters and number of iterations in OSEM (Hanning filter with a cut-off of 0.85 and 8 subsets and 4 iterations). Iterative Reconstruction Attenuation Corrected with applied 3D post filtering (IRACF) was used as the nomenclature for iteratively reconstructed SPECT data in this study.

A programme was developed to calculate the ACF of the reconstructed PET images, performed both in frequency and spatial domain. The spatial equation is based on 2D cross-correlation of the matrix:

*a*_*i,,j *_with image resolution (image size) of *i *× *j *with itself using the lags *k *and *l*



where *k *and *l *refer to lags of the function and *m *and *n *refer to the number of steps needed for performing the masking and

max(1,1 - *k*) ≤ *i *≤ min(*m*,*m *- *k*)

and

max(1,1 - *l*) ≤ *j *≤ min(*n*,*n *- *l*)

To avoid disturbing effects at the edges of the FOV an arbitrary central slice within each frame was used. Subsequently, an arbitrary chosen matrix with a size of 25 × 25 from the central part of the slice was used as a mask when applying the ACF. After subtraction of the average over this matrix, the ACF image was generated showing the correlation of the noise in the image. The resulting image was then normalized by dividing each pixel value by the maximum pixel value within the generated ACF image. The results from this procedure applied to images from all experiments were studied and compared.

The aim of the ACF application was to describe the noise correlation between the pixels within each image. A specific aim was to analyse the form and the shape of the 2D autocorrelation function in the images in different imaging modalities. The programme results in images, which can be used for the visualization and comparison of images using different techniques obtained with different reconstruction algorithms and for plotting 1D vertical and horizontal profiles through the images.

Another programme was developed to calculate the variance matrix as indicator of the noise magnitude distribution across the image plane. The method compares pixel values through several adjacent slices within a frame and calculates



where *n *is the total number of slices used, *i *scans through pixels in-between slices and *j *defines a position within the two-dimensional image matrices. E.g., in PET, *j *spans from 1 to 128 × 128. The aim of this application was to study the magnitude of noise in relation to position within the images produced by different modalities. The results are illustrated and studied as 2D variance images and 1D horizontal profiles across the images.

## Results

### PET studies

**Figure 1:**
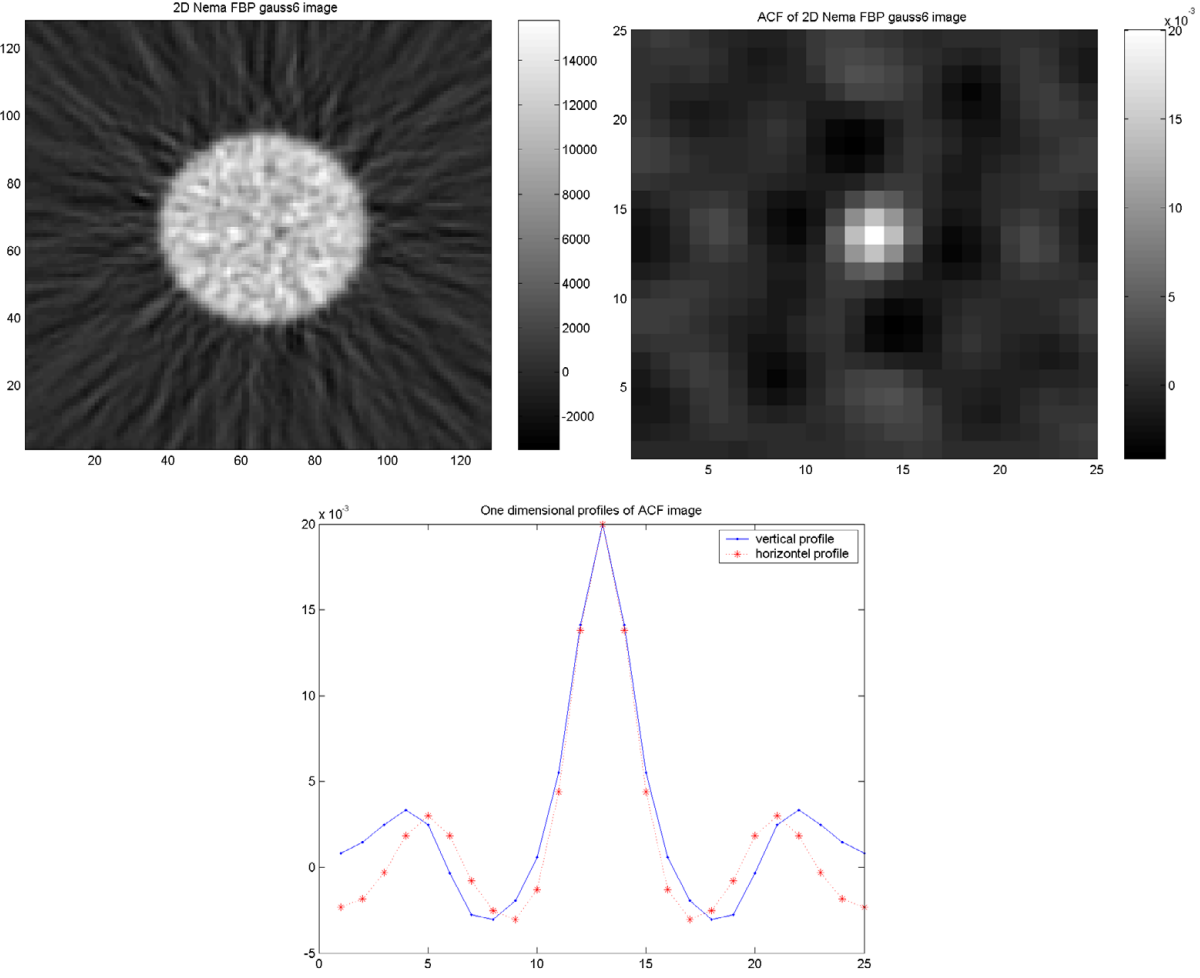
Results of the cylindrical NEMA phantom study. Acquired image (upper left) reconstructed using FBP with applied 6 mm (FWHM) Gaussian filter, corresponding ACF image (upper right) and vertical (dash point) and horizontal profiles (dash star) through the ACF image.

**Figure 2:**
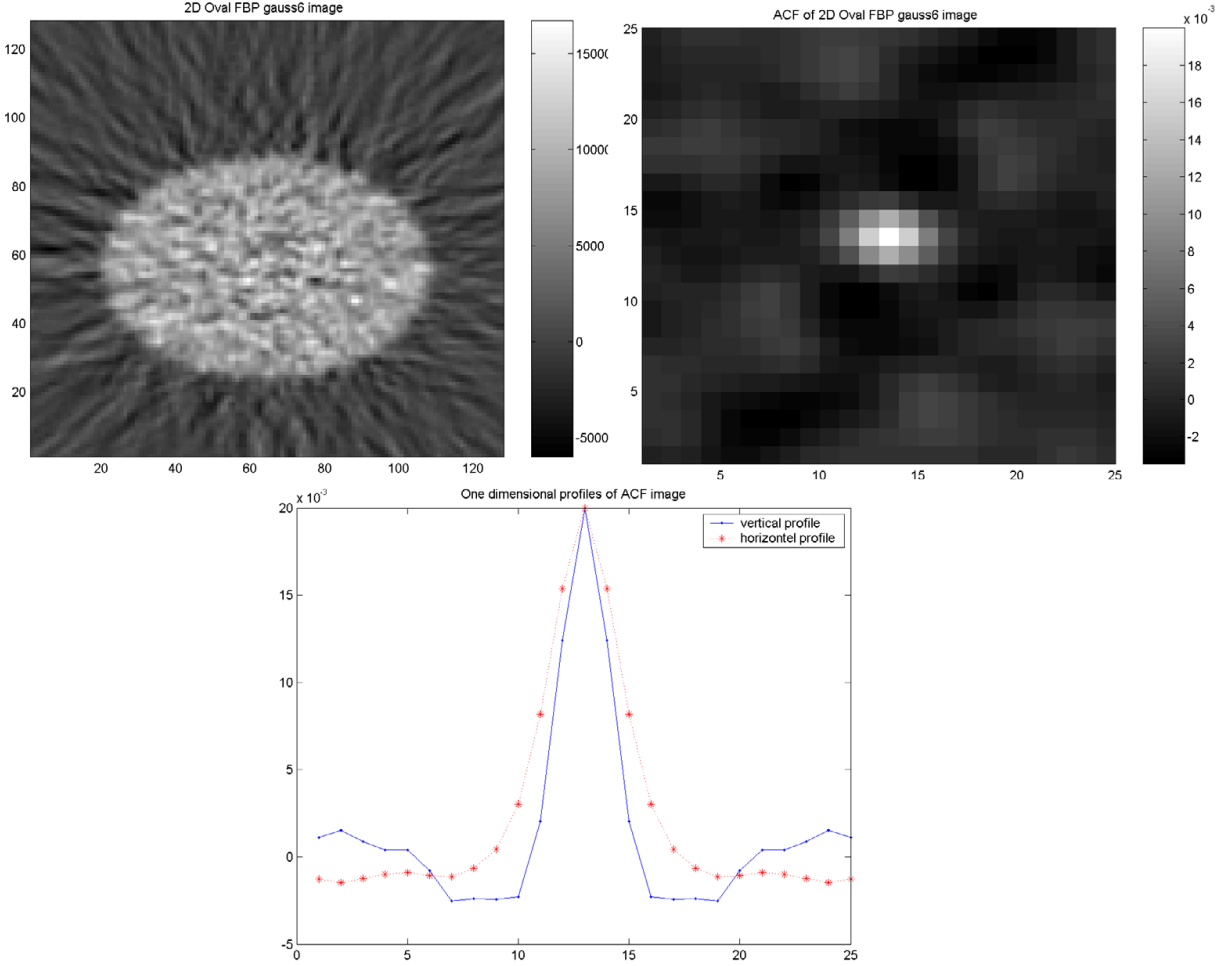
Results of the elliptical torso phantom study. Image (upper left) reconstructed using FBP with applied 6 mm (FWHM) Gaussian filter, corresponding ACF image (upper right) and vertical (dash point) and horizontal profiles (dash star) through the ACF image.

**Figure 3:**
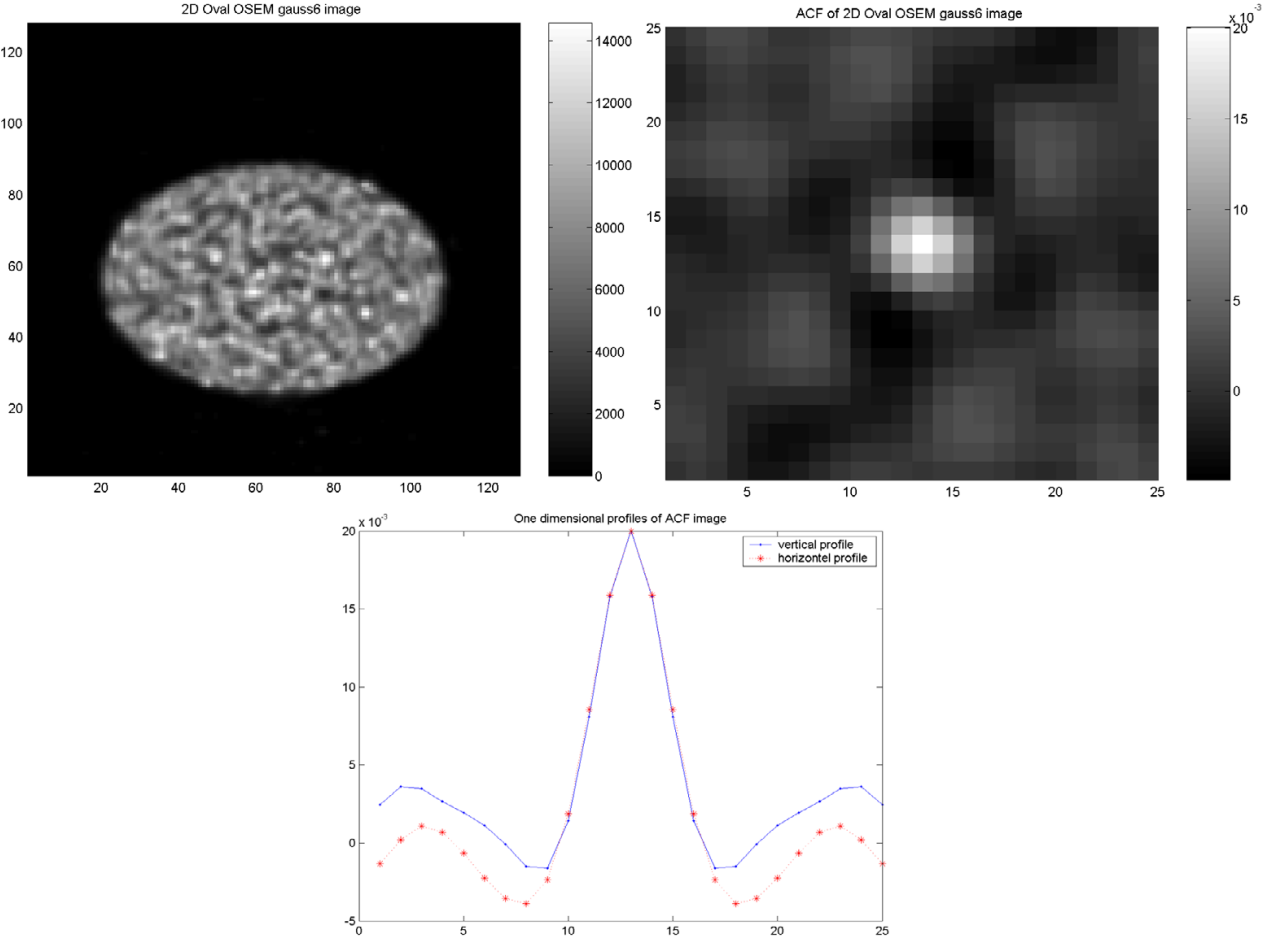
Results of the elliptical torso phantom study. Image (upper left) reconstructed using OSEM with applied 6 mm (FWHM) Gaussian filter, corresponding ACF image (upper right) and vertical (dash point) and horizontal profiles (dash star) through the ACF image.

In the study on the NEMA phantom (Figure 1), the results indicate an identical and isotropic ACF with a similar pattern of noise texture independent of applied reconstruction methodology and used filter (6 mm Gaussian and 4 mm Hanning produced identical results). In the torso phantom (Figure 2), however, the results of the ACF indicate a non-isotropic correlation of the noise in images reconstructed using FBP, independent of used filter (6 mm Gaussian and 4 mm Hanning). On the other hand, the images reconstructed using OSEM (Figure 3), show an identical and isotropic form with a similar pattern of noise texture independent of used filter.

### CT Studies

**Figure 4:**
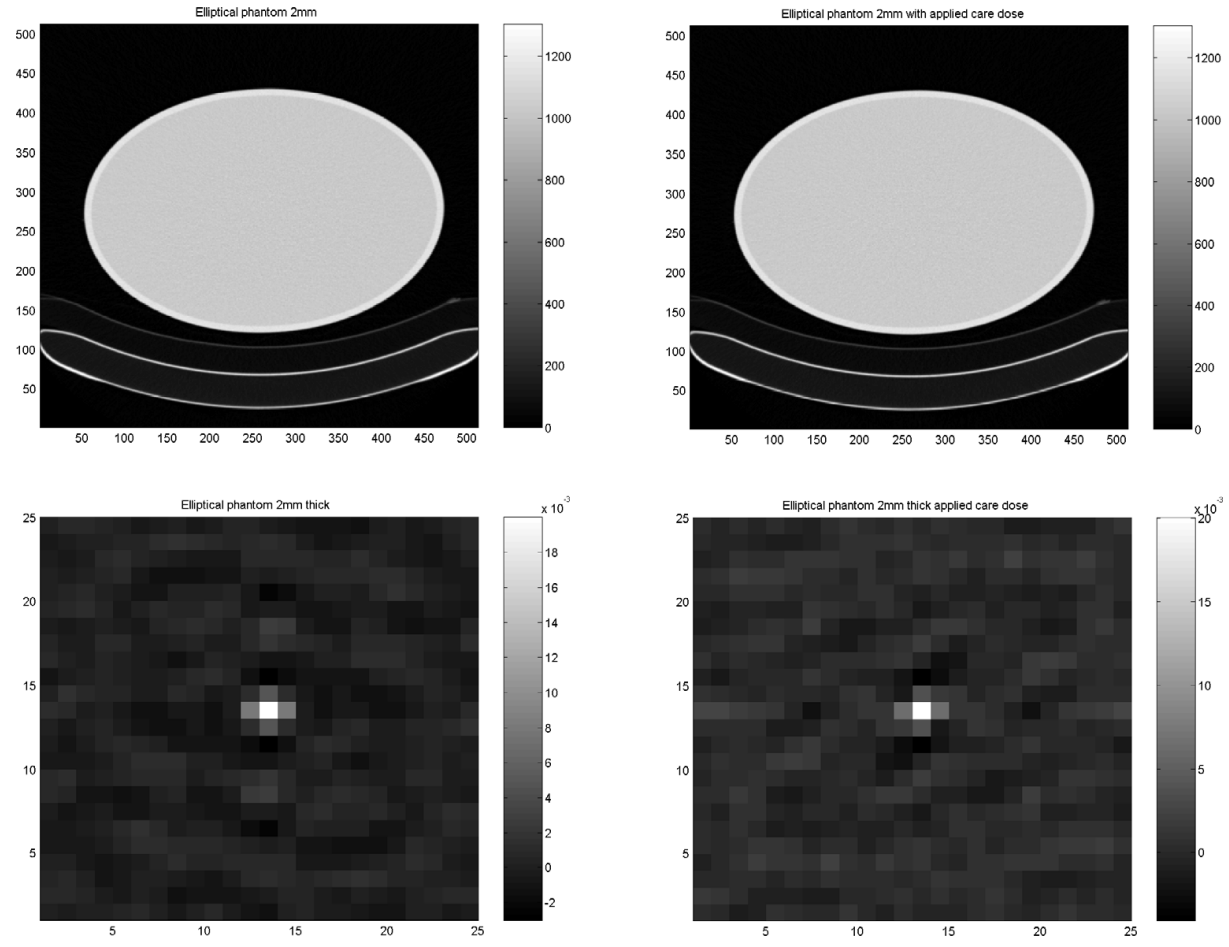
Results of the cylindrical Torso phantom in CT study. FBP image with thickness of 2 mm scanned with constant mA setting (upper left) respective with applied care dose (upper right). ACF images from the normal scan (lower left) respective with applied care dose (lower right).

**Figure 5:**
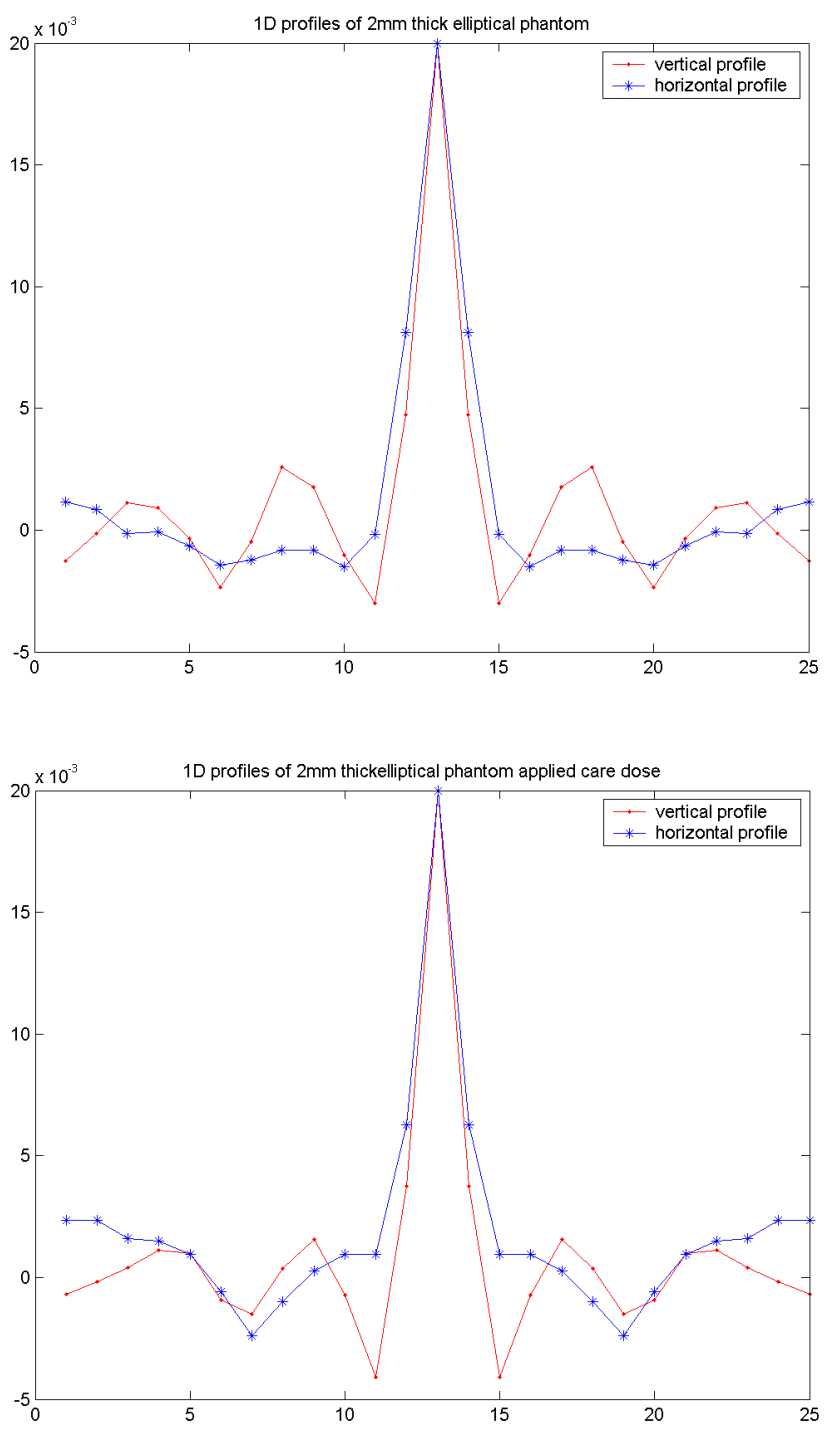
1D vertical and horizontal profiles through the ACF image based on the normal scan (upper) respective with applied care dose (lower).

In CT images of the circular Nema phantom, the ACF shows the expected isotropic behaviour. When the elliptic torso phantom was scanned, a slightly non-isotropic behaviour was observed as indicated by the ACF (Figures 4, 5). This non-isotropy is only slightly affected by the use of the care dose. Identical results were obtained with 2- and 3-mm slice thickness.

### SPECT studies

In the studies of noise autocorrelation in SPECT images, the images from the cylindrical phantom showed close to but not entirely isotropic behavior.

**Figure 6:**
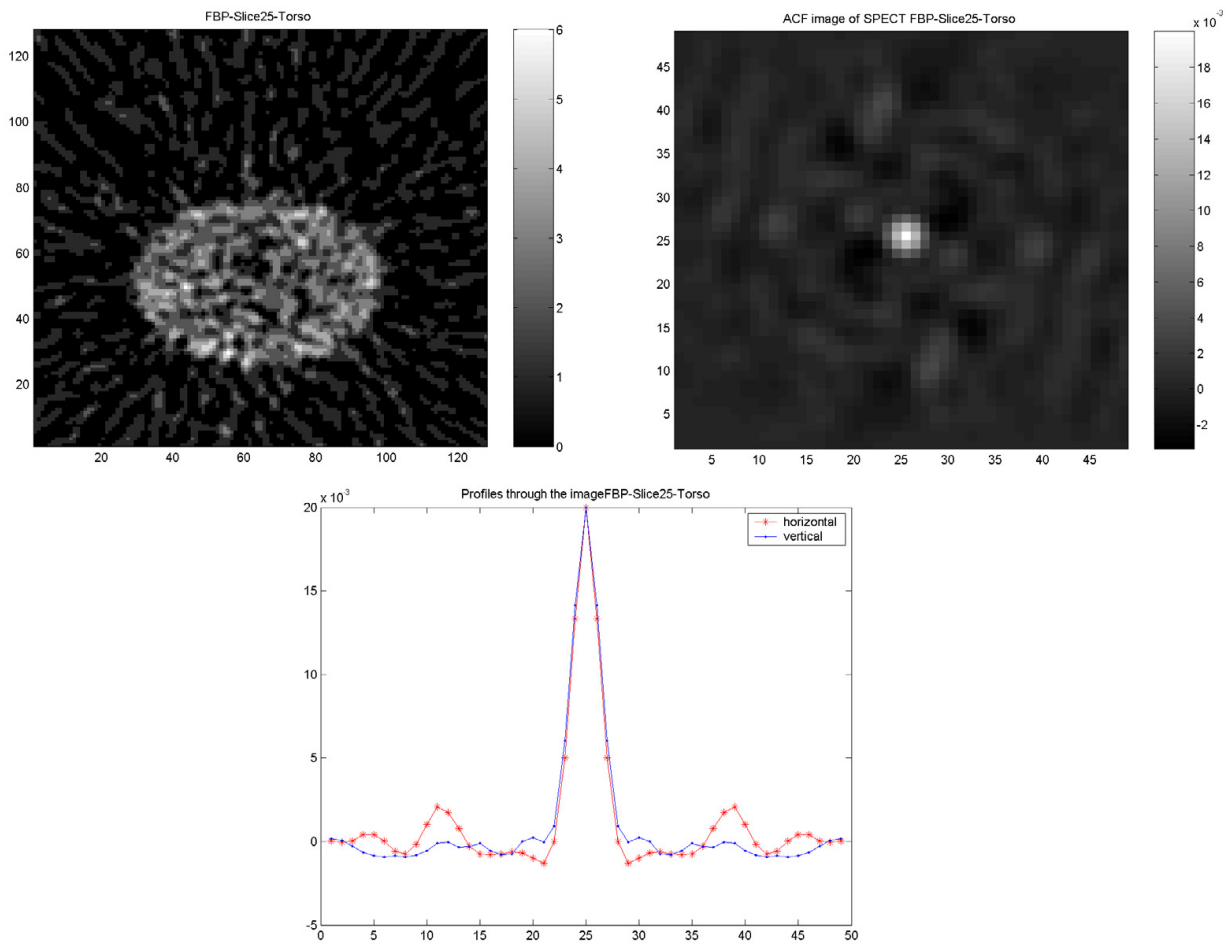
Results of SPECT study on elliptical Torso phantom. Image reconstructed using FBP followed by 3D filtering (upper left) and corresponding ACF (upper right) followed by 1D vertical and horizontal profiles of the ACF (down).

Images from the elliptic phantom showed an ACF which was close to isotropic (Figure 6). The same was true when the images were reconstructed with the IRACF iterative reconstruction.

### PET/CT studies

**Figure 7:**
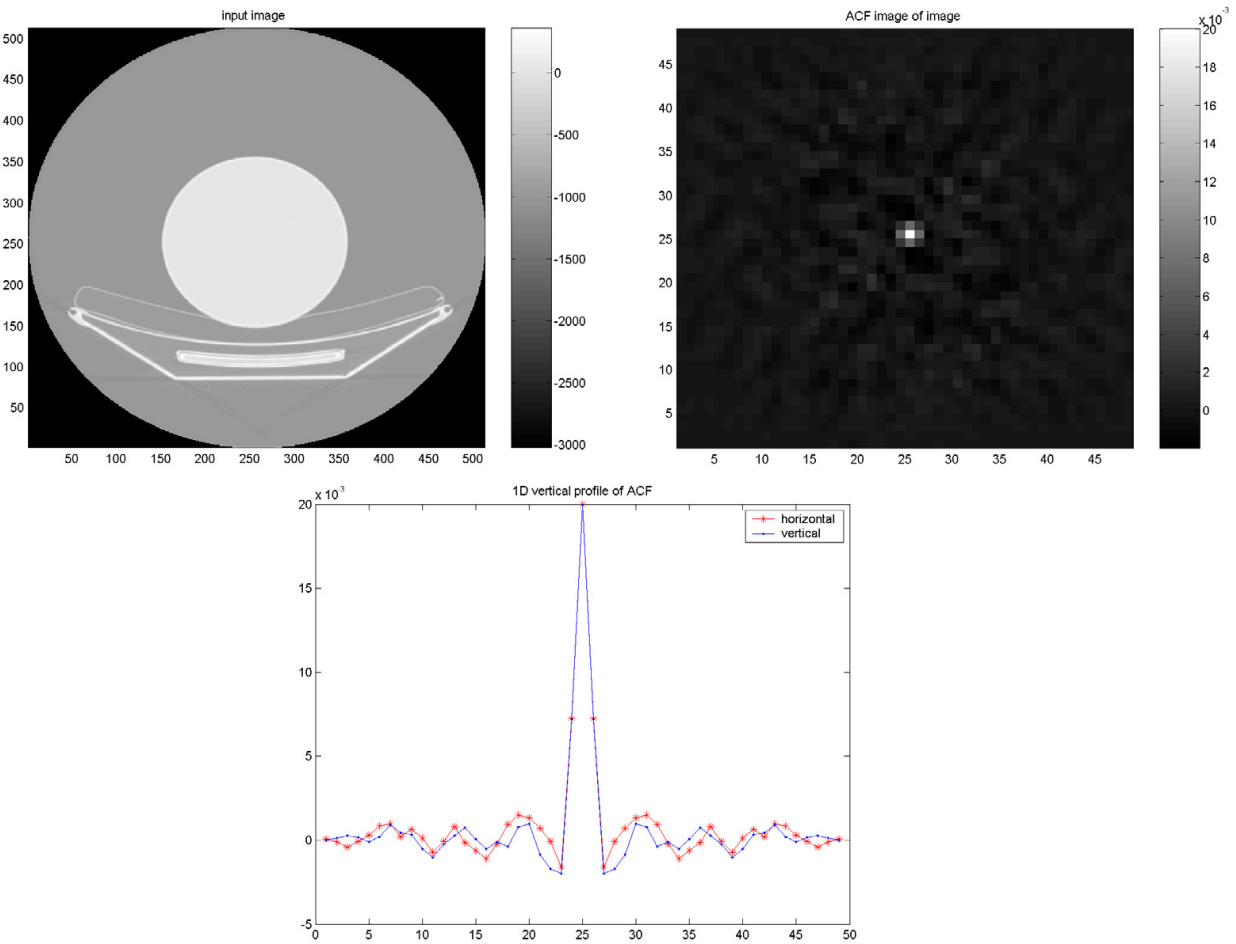
Results of PET/CT study on the cylindrical Nema phantom. The CT image reconstructed using FBP (upper left) and corresponding ACF (upper right) followed by 1D vertical and horizontal profiles of the ACF (down).

**Figure 8:**
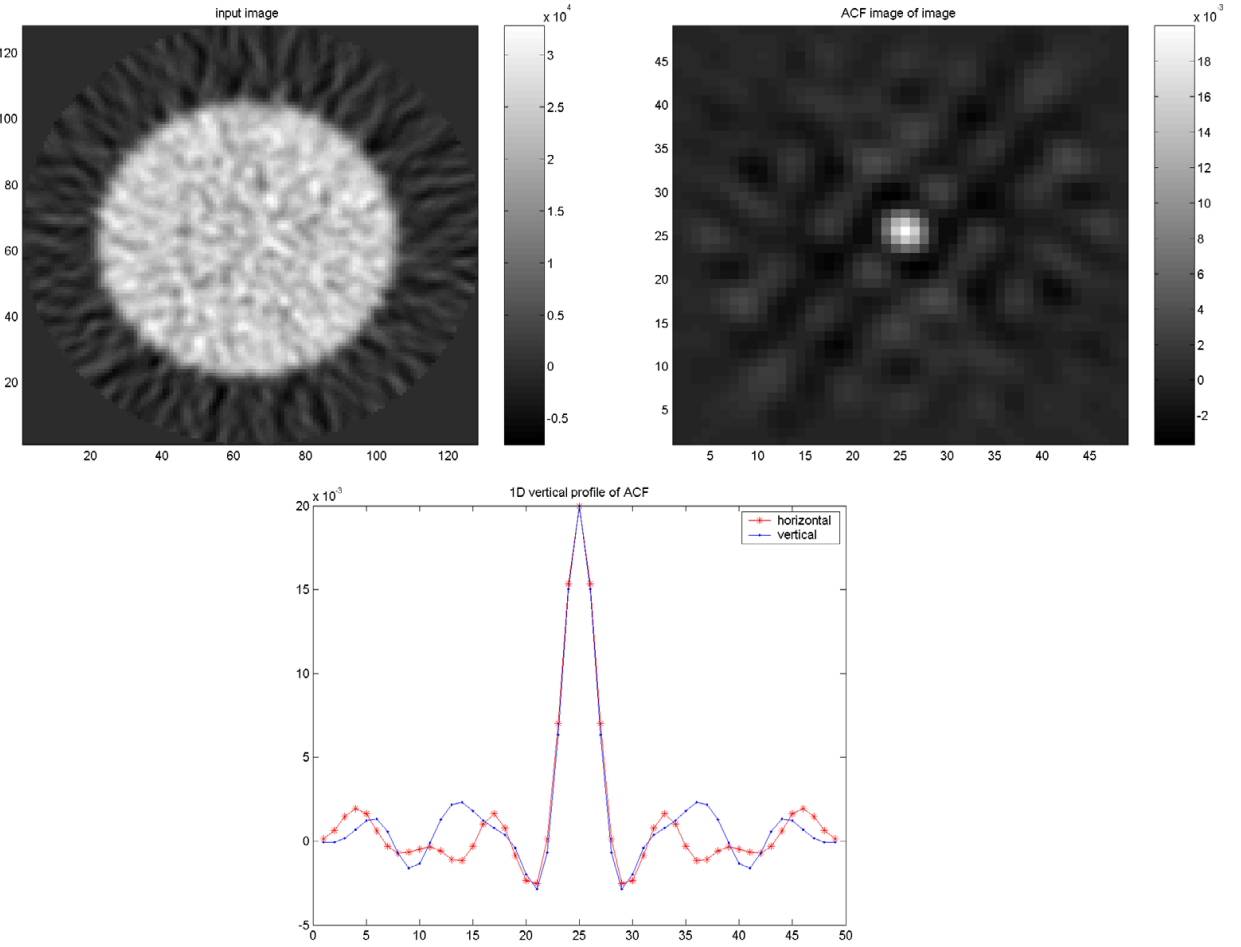
Results of PET/CT study on the cylindrical Nema phantom. The PET image reconstructed using FBP with applied 4-mm Hanning filter (upper left) and corresponding ACF (upper right) followed by 1D vertical and horizontal profiles of the ACF (down).

**Figure 9:**
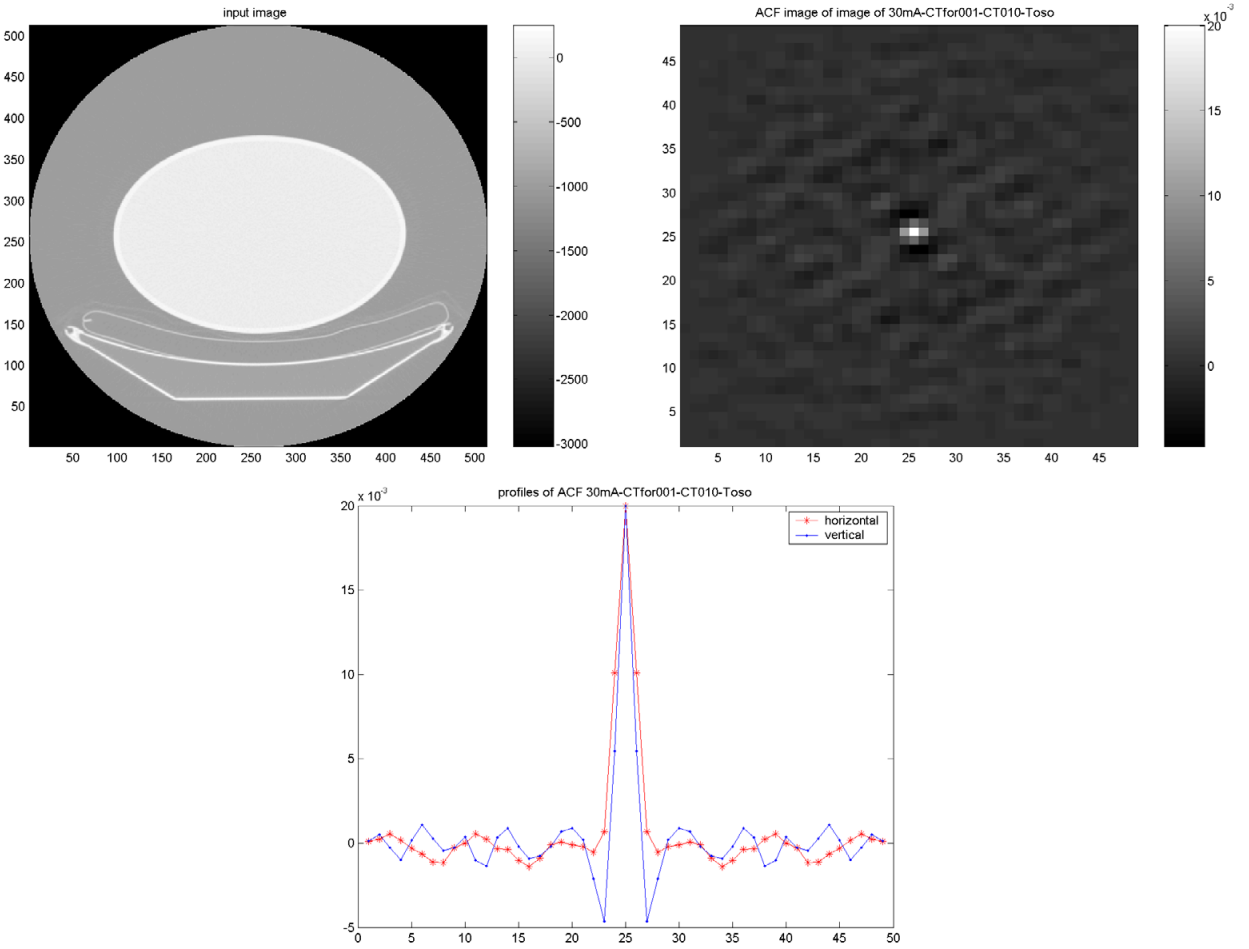
Results of PET/CT study on the elliptical Torso phantom. The CT image reconstructed using FBP (upper left) and corresponding ACF (upper right) followed by 1D vertical and horizontal profiles of the ACF (down).

**Figure 10:**
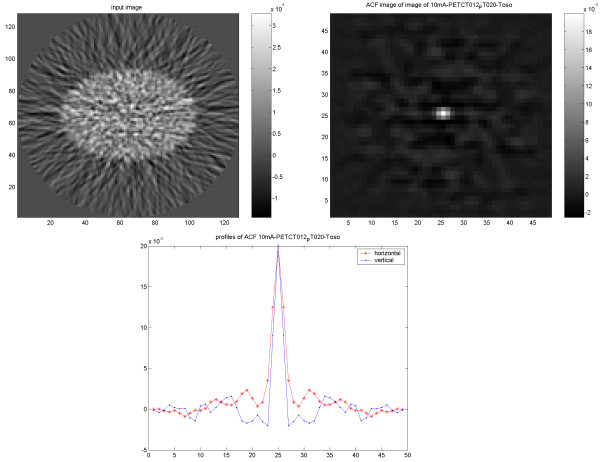
Results of PET/CT study on elliptical Torso phantom. PET image reconstructed using FBP with applied 4-mm Hanning filter (upper left) and corresponding ACF (upper right) followed by 1D vertical and horizontal profiles of the ACF (down).

**Figure 11:**
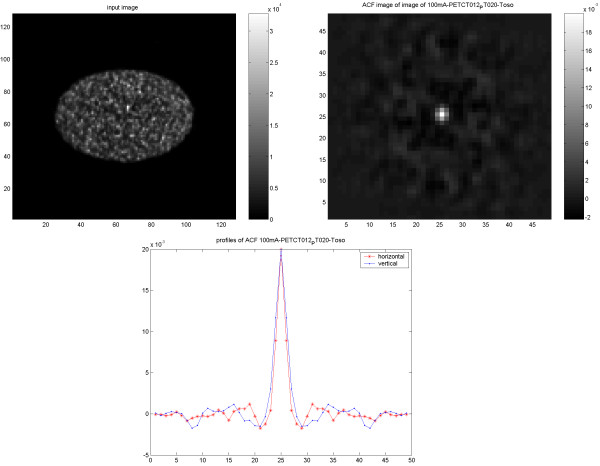
Results of PET/CT study on elliptical Torso phantom. PET image reconstructed using OSEM with applied 4-mm Hanning filter (upper left) and corresponding ACF (upper right). 1D vertical and horizontal profiles of the ACF (down).

The CT images (Figure 7) and the PET images (Figure 8) from the cylindrical phantom using PET/CT showed an expected isotropic behavior, which in the case of PET was independent of reconstruction method. When scanning and reconstructing images from the elliptic phantom; however, both the CT images (Figure 9) and PET images (Figure 10) showed a slightly non-isotropic behavior. When the PET image was reconstructed using OSEM, the noise correlation became more isotropic (Figure 11). There was an identical noise correlation pattern in the PET images reconstructed with different CT doses.

### Variance images

**Figure 12:**
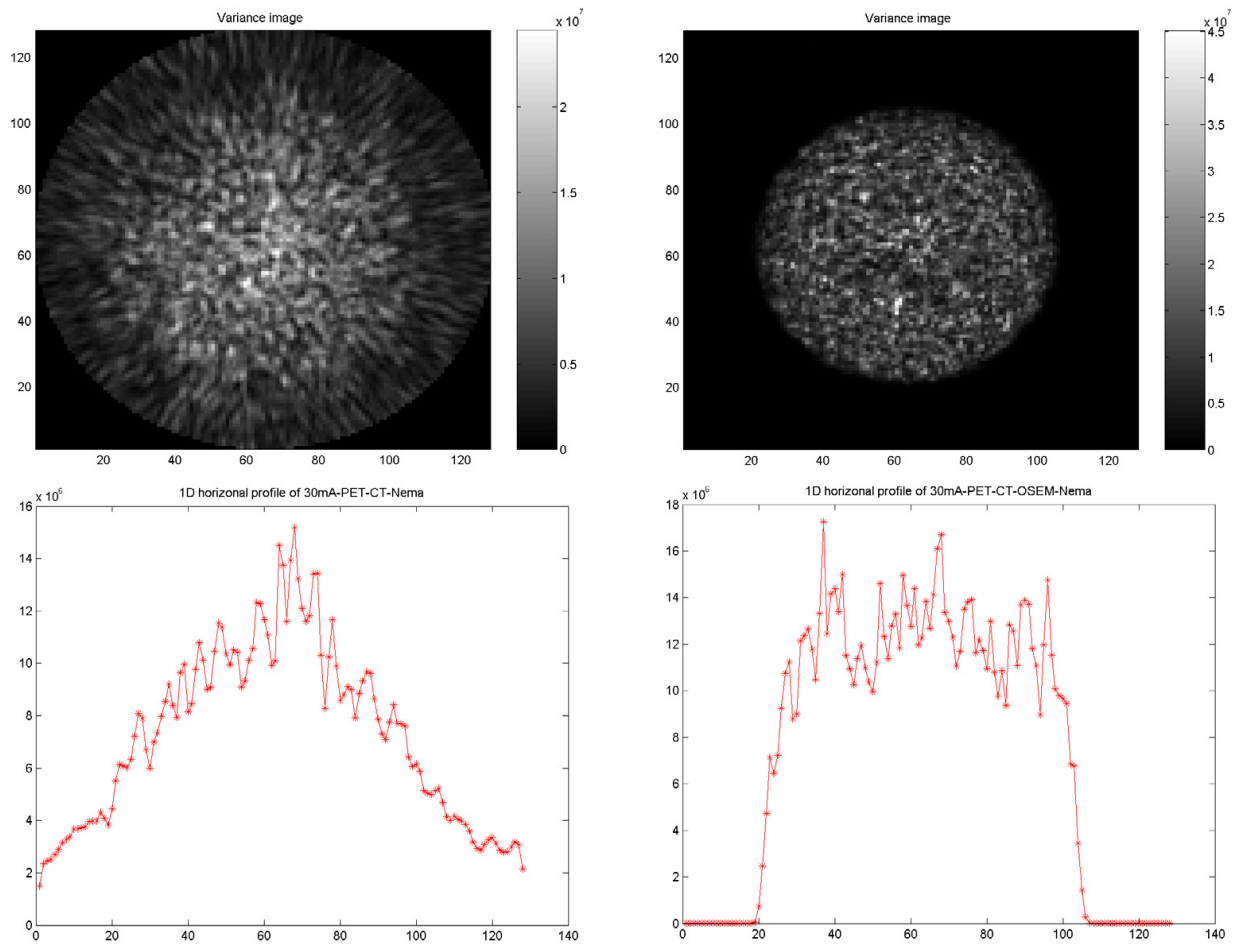
PET results of PET/CT study on cylindrical NEMA phantom. Variance images reconstructed using FBP (upper left) and OSEM (upper right). 1D horizontal profile through the variance image reconstructed using FBP (lower left) and reconstructed using OSEM (lower right).

**Figure 13:**
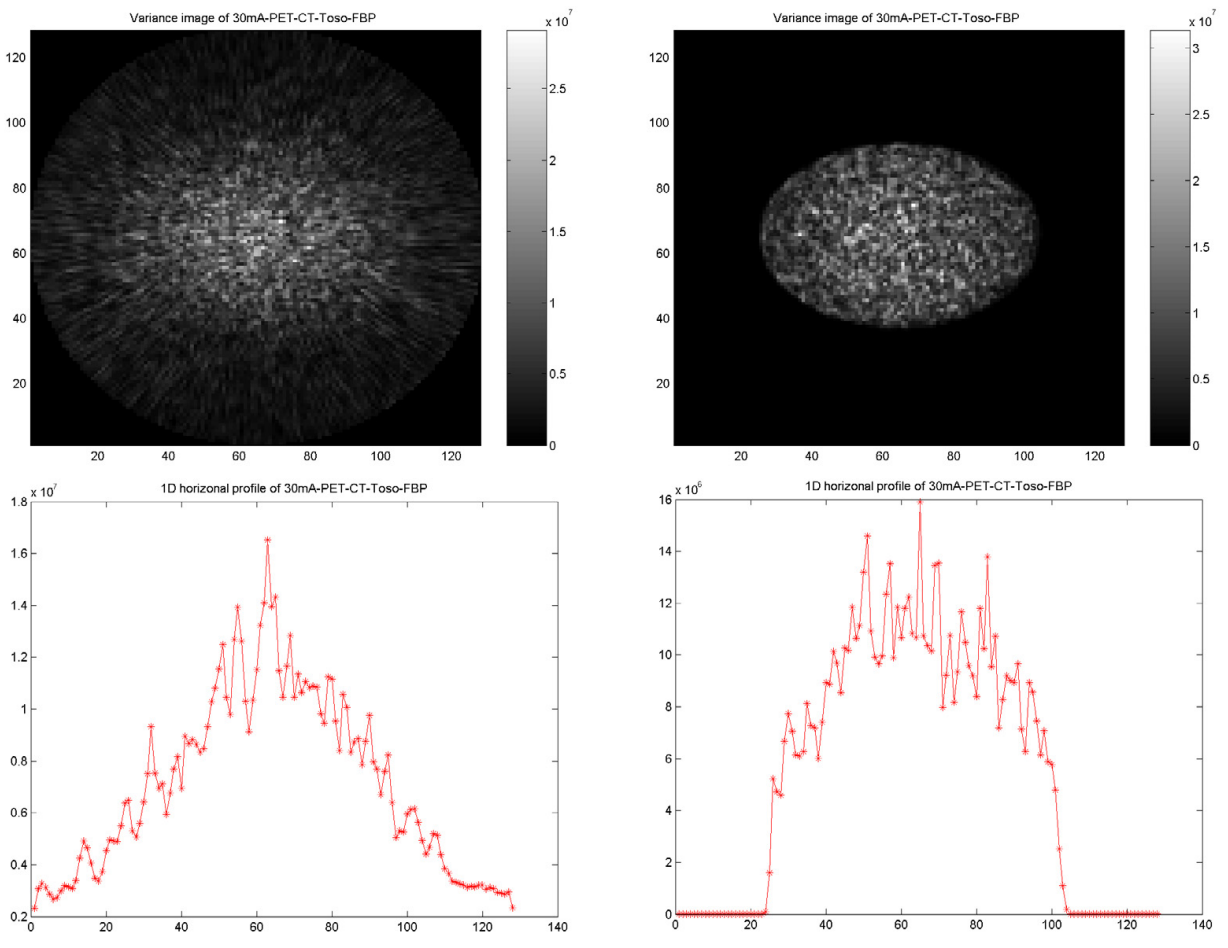
Results of PET/CT study on elliptical Torso phantom. PET variance images reconstructed using FBP (upper left) and OSEM (upper right). 1D horizontal profile through the variance image reconstructed using FBP (lower left) and reconstructed using OSEM (lower right).

Variance images were generated from PET, SPECT, CT and PET/CT images reconstructed using FBP and OSEM for further illustration of differences in noise behavior. The results obtained from images using the same type of reconstruction method showed similar features, independent of the used imaging modality. In PET and PET/CT reconstructed with FBP the noise variance gradually decreased from inside to outside of the phantom, whereas with OSEM, the noise decreased abruptly at the border of the object (Figures Figure 12, 13).

**Figure 14:**
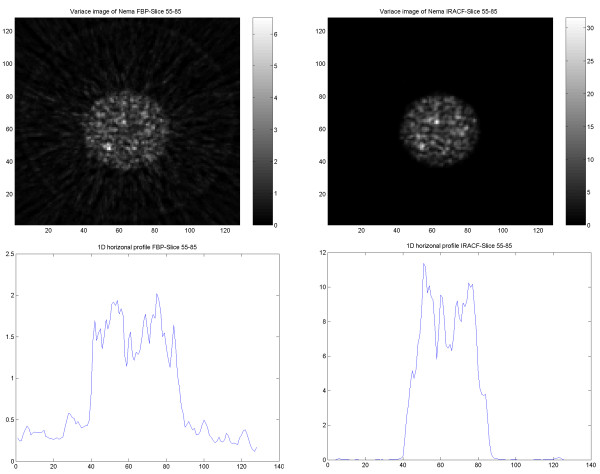
Results of the SPECT study on the cylindrical NEMA phantom. Variance image reconstructed using FBP (upper left) and IRACF (upper right). 1D horizontal profile through the variance image reconstructed using FBP (lower left) and reconstructed using IRACF (lower right).

**Figure 15:**
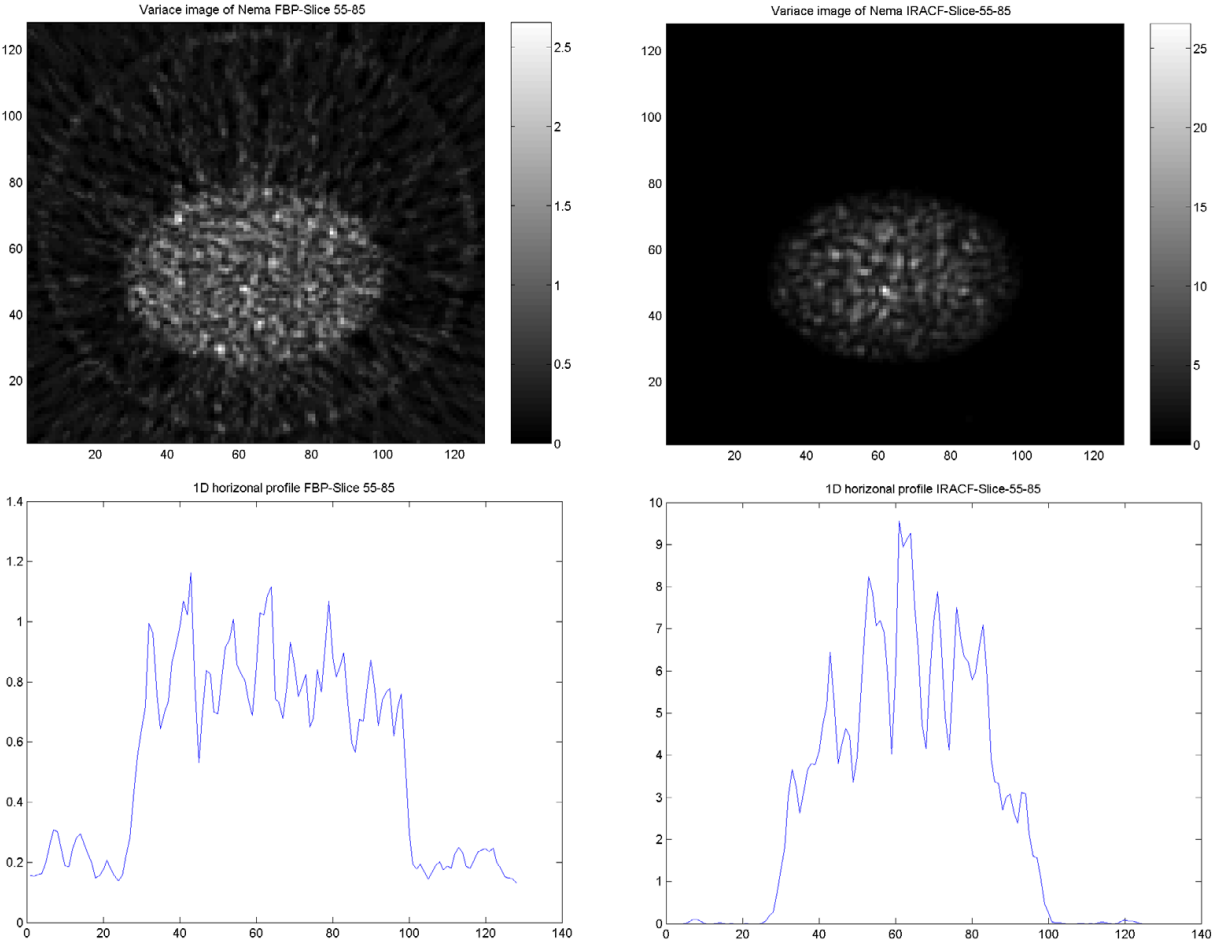
Results of the SPECT study on elliptical Torso phantom. Variance image reconstructed using FBP (upper left) and IRACF (upper right). 1D horizontal profile through the variance image reconstructed using FBP (lower left) and reconstructed using IRACF (lower right).

In the SPECT images, the noise magnitude in the FBP images decreased more abruptly at the border of the object, thereby resembling images reconstructed by IRACF (Figures Figure 14, 15).

**Figure 16:**
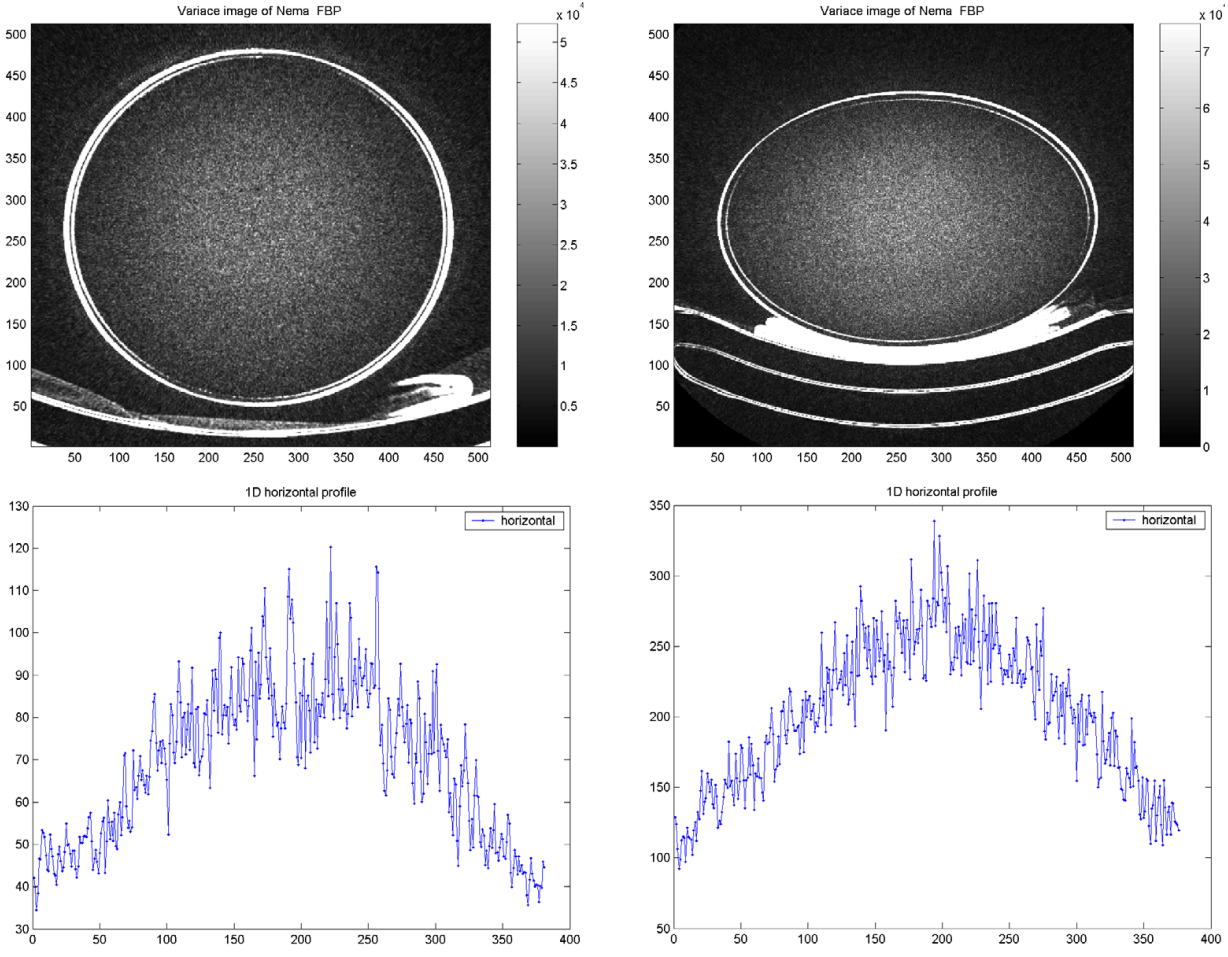
Variance across the CT image of the cylindrical (left) and elliptic (right) phantom. Horizontal profiles over the corresponding variance images are shown in the graphs.

**Figure 17:**
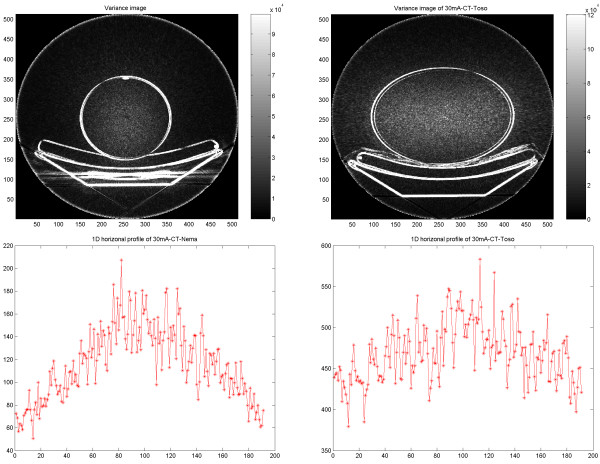
Results of the PET/CT study on cylindrical NEMA phantom. Variance images of CT (transmission scan) of Nema (upper left) and Torso phantom (upper right). Corresponding 1D horizontal profile through the variance images of Nema (lower left) and Torso phantom (lower right).

The variance across the CT image field showed a broad maximum at the centre of the object and gradually decreased towards the periphery. The same features were observed in the cylindrical and the elliptic phantom. The variance at the centre was about three times higher than that at the periphery. Similar behavior was observed independently of slice thickness and application of care dose (Figure 16). Figure 17 visualizes the noise distribution over the image field for the CT in PET/CT. The noise was significantly higher in the centre of the cylindrical phantom, similar to that observed in the previous study with CT. With the cylindrical phantom the variation along the horizontal axis was less than along the vertical axis. Almost identical results were obtained using different magnitude of X-ray dose.

### Sinogram data in PET and SPECT

**Figure 18:**
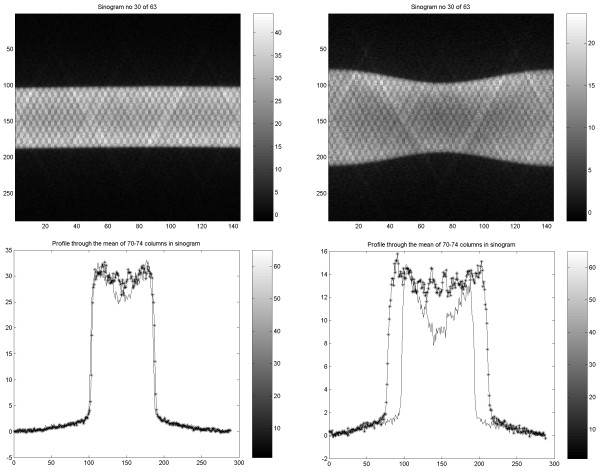
Sinograms from PET study of Nema (left) and Torso (right) phantom illustrating detector counts for the different projections (upper images). Profiles through the end and central parts of the sinograms, corresponding to horizontal vs. diagonal projections during acquisition (lower plots).

To understand the noise contribution, profiles were generated across the sinograms from PET studies with the two phantoms. The counts recorded from the circular phantom dipped slightly in the central part (Figure 18). For the elliptic phantom this central dip was more accentuated in the plot over the short axis representation but the profile was rather flat in its central portion for the long axis representation.

**Figure 19:**
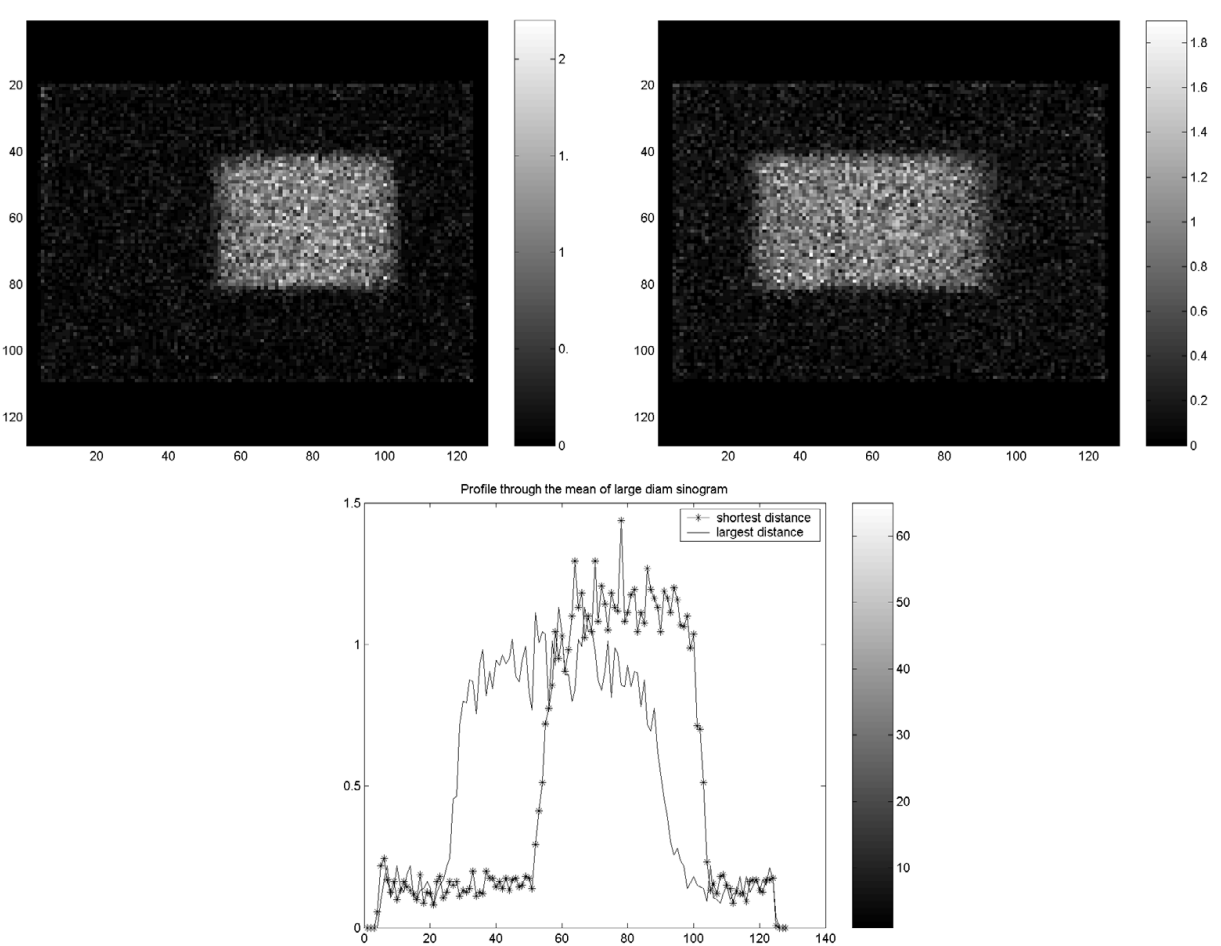
Projections obtained at two perpendicular angles at the acquisition with SPECT for the Torso phantom. Profiles through the two different projections, corresponding to horizontal vs. diagonal direction in SPECT acquisition (down). The position shift is related to the fact that the phantom was not centred at the rotation axis.

The corresponding plots for SPECT showed a different behavior, with higher counts centrally and rather similar count values for the two projections in the elliptic phantom (Figure 19).

## Discussion

The aim of the present study was to explore and compare the properties of noise, notably its correlation, in images from CT, PET, PET/CT and SPECT. One of the main focuses was to illustrate the differences in noise correlation between images reconstructed using different types of reconstruction algorithms such as FBP and OSEM, generated utilizing different imaging modalities. We believed that despite their similarity as tomographic imaging devices, the fact that the acquisition of detector data and modes of reconstruction differ could lead to differences in the expression of noise in the images. CT is based on measurement of transmitted X-ray photons from X-ray tube through the object and to the detectors. PET is based on the simultaneous measurement of two annihilation photons that emerge from the body and hit each of two detectors on opposite sides of the object. SPECT is based on recording single photons that emerge from the body and acquisition of a large number of projections by sequential rotation of the detector system around the body. For these techniques the measured data, typically organised in projections are subjected to different corrections, where especially in PET and SPECT the attenuation correction is dominant. After performing the different corrections the projection data are utilised in a reconstruction algorithm for the generation of images. The reconstruction part can be similar for the three devices with the predominant methods being FBP or iterative reconstruction.

Since noise in the images is a factor which may impair the visualisation of discrete signals and the generation of quantitative values, it is important to understand its features. To illustrate some aspects of noise we developed a programme to generate auto-correlation images which predominantly indicate the noise correlation and the angular dependence of noise correlation. Images depicting the variance across the image planes were created to indicate the spatial dependence of noise. Some of the characters of noise were explained by studying the profiles of counts in different directions in the sinogram domain in PET and SPECT.

Applying the auto-correlation function on the reconstructed PET, PET/CT and SPECT data revealed a clear correlation: the noise was affected by the adjacent 2–3 pixels corresponding to 4.2–6.3 mm in PET, 4.7–7.0 mm in PET/CT and 8.9–13.4 mm in SPECT. This correlation was similar for all devices and all reconstruction algorithms. As expected, an isotropic pattern of correlation of the noise in the images was obtained in the circular Nema phantom. In CT scans from both regular CT and PET/CT, the same behaviour was observed. The radial dependence of noise correlation is highly dependent upon reconstruction algorithm, and ensuing spatial resolution which especially with OSEM in a complicated manner is depending on number of iterations and shape and distribution of the object. Hence in the present work, the width of the autocorrelation function per see can not be generalised, but the concept that the autocorrelation function may have a non-isotropic shape.

Hence we observed that the noise correlation pattern obtained from the torso phantom with elliptical shape was clearly non-isotropic when examined with PET or PET/CT and dissimilar between images using the two reconstruction methods. When studies were performed on this non-circular phantom, ACF images from PET, CT and PET/CT studies reconstructed using FBP showed an asymmetric correlation texture of the noise. This non-isotropic behaviour can be explained in three different ways; the first explanation is that the relative noise in sinogram data in the study on Torso phantom differs in different directions. The sinogram data indicate that the count rates are higher in the direction of the shortest diameter compared to the angle with the longest diameter. This is because along the 30 cm axis the detectors see a 50 % higher amount of radioactivity. However, this radioactivity is in turn subjected to an attenuating path that is 10 cm longer, leading to an overall 43% lower count rate. Since noise in the projection data is Poisson distributed, the noise standard deviation is angular dependent and 25% higher along the long axis of the phantom. The second explanation is related to the attenuation correction whereby the projection data with its noise is multiplied by attenuation correction factors which are different for different projections. The third contribution is related to the way noise is handled in the FBP algorithm when the noise magnitude differs in the different projections. The noise modification and correlation induced by the convolution filter in the reconstruction is the same in all projections. The back projection then distributes noise with different magnitude in different angular directions.

The correlation pattern of the noise in images from PET, CT and PET/CT studies become close to symmetric when the data are reconstructed using OSEM. This behaviour depends on how the iterative reconstruction algorithm handles the noise with an inherent attempt to iterate to similar deviations for each angular projection, which then tends to equalise the noise magnitude for the different projections.

In PET/CT studies the results from fused images were identical independent of the applied dose of X-ray in transmission part (CT). This observation indicates that the noise from the transmission at these doses does not propagate into the fused images because the noise from the emission part is dominant. The result suggests that in human studies it might be possible to use a low dose in the CT transmission part while maintaining the noise behaviour and the quality of the images. This observation supports the outcomes of the study done by Kamel et al [30] evaluating the effect of lowering the CT tube current in human PET/CT studies and suggesting that a lower X-ray dose to the patient could be used without impairment of image quality.

In SPECT studies, the correlation pattern was still close to isotropic in images obtained from an elliptical object and independent of the reconstruction methodology used. This behaviour could be because the number of counts in different directions in the study on either circular or elliptical phantom is almost the same. This similarity is caused by the way radioactivity is sensed, with a detector which records the superficial activity and not that deep inside the object. Since attenuation is a dominant factor with respect to recording of radioactivity, the number of counts will become relatively similar for a uniform elliptic phantom. On the other hand, a focal radioactivity not centred in the phantom will instead give highly variable counts in different detectors. Additionally the detector sensitivity is significantly affected by distance from the detector. This could explain a slightly non-isotropic effect observed in the circular phantom that was not perfectly centred.

ACF images from CT studies reconstructed by FBP on data from the Torso phantom showed a non-isotropic form. The correlation pattern was broader along the horizontal axis compared with the vertical. This non-isotropic behaviour depends on how data is acquired in CT. The X-ray tube rotating around the object emits X-rays, which are detected by detectors that are placed as a block on the opposite side of the tube. The number of photons detected is highly dependent upon the thickness of the object, and the relative noise is hence larger in the horizontal direction of the object. These differences in noise magnitude are, as indicated above, handled by the FBP algorithm such that a non-isotropic noise correlation is given in the images.

The results from studies on CT images with applied care dose function gave slightly different noise correlation compared to those without care dose. Yet the noise correlation was not identical in the vertical and horizontal direction, suggesting that the care dose could not fully equalise the relative noise in different directions.

The variance across the PET images shows a significantly broader distribution with FBP than with OSEM. With OSEM, the variance reduces abruptly at the border of the object. The variance is significantly larger centrally in the object than at the periphery.

## Conclusion

The combined effect of noise correlation for asymmetric objects and a varying noise variance across the image field significantly complicates the interpretation of the images when statistical methods are used, such as with statistical estimates of precision in average values, use of statistical parametric mapping methods and principal component analysis. Hence it is recommended that iterative reconstruction methods are used for such applications. However, it is possible to calculate the noise analytically in images reconstructed by FBP, while it is not possible to do the same calculation in images reconstructed by iterative methods. Therefore for performing statistical methods of analysis which depend on knowing the noise, FBP would be preferred.

## Competing interests

The author(s) declare that they have no competing interests.

## Authors' contributions

Authors PR and MB designed the study. They created the method for applying ACF, performed the image and data analysis and drafted the paper.

Author MS helped perform the SPECT studies, acquire and reconstruct the data and write this paper.

Authors HS, EB, EM and BL helped with some practical approaches and the writing of this paper.

## Pre-publication history

The pre-publication history for this paper can be accessed here:


